# Tripeptide Self-Assembly into Bioactive Hydrogels: Effects of Terminus Modification on Biocatalysis

**DOI:** 10.3390/molecules26010173

**Published:** 2020-12-31

**Authors:** Marina Kurbasic, Ana M. Garcia, Simone Viada, Silvia Marchesan

**Affiliations:** Chemical & Pharmaceutical Sciences Department, University of Trieste, 34127 Trieste, Italy; marina.kurbasic@studenti.units.it (M.K.); anamariagarcia.1988@gmail.com (A.M.G.); simone.viada@studenti.units.it (S.V.)

**Keywords:** peptides, hydrogels, biomaterials, self-assembly, chirality, d-amino acids, hydrolase, biocatalysis

## Abstract

Bioactive hydrogels based on the self-assembly of tripeptides have attracted great interest in recent years. In particular, the search is active for sequences that are able to mimic enzymes when they are self-organized in a nanostructured hydrogel, so as to provide a *smart* catalytic (bio)material whose activity can be switched on/off with assembly/disassembly. Within the diverse enzymes that have been targeted for mimicry, hydrolases find wide application in biomaterials, ranging from their use to convert prodrugs into active compounds to their ability to work in reverse and catalyze a plethora of reactions. We recently reported the minimalistic l-His–d-Phe–d-Phe for its ability to self-organize into thermoreversible and biocatalytic hydrogels for esterase mimicry. In this work, we analyze the effects of terminus modifications that mimic the inclusion of the tripeptide in a longer sequence. Therefore, three analogues, i.e., *N*-acetylated, *C*-amidated, or both, were synthesized, purified, characterized by several techniques, and probed for self-assembly, hydrogelation, and esterase-like biocatalysis. This work provides useful insights into how chemical modifications at the termini affect self-assembly into biocatalytic hydrogels, and these data may become useful for the future design of supramolecular catalysts for enhanced performance.

## 1. Introduction

The ability of natural enzymes to catalyze reactions with exquisite selectivity in water has been inspiring scientists for a long time in the search for simpler, synthetic mimetics that are ultimately biodegradable for a low impact on the environment [1]. In the area of biomaterials, enzyme mimicry has found a wide variety of applications to tackle unsolved challenges, ranging from combating biofilms [2] to cancer therapy [3]. In particular, hydrolases are among the most studied targets to develop mimetics thanks to their robustness and ability to catalyze a wide range of reactions [4,5]. They have been anchored on biopolymers for enhanced activity and durability [6], or they have been used for the formation of products for biomedical use [7]. A popular strategy includes hydrolase-sensitive bioactive molecules in the biomaterial to make it adaptive to the biological milieu, for tissue engineering [8], immunomodulation [9], or antimicrobial activity [10], for instance.

One shortcoming of enzymes, however, is that they typically comprise over one hundred amino acids and may even trigger an immune response; thus, it is not surprising that the search has been very active to replace them with shorter sequences [11]. Self-assembling peptides appear ideal candidates thanks to the possibility to encode catalytic functions of the enzyme directly into the peptide sequence, combined with the ability to form supramolecular hydrophobic pockets for reactions to occur, as well as the added value of multivalency of the resulting (nano)structures [12]. However, their design is notoriously challenging, especially in light of the diverse conformations they can adopt [13]. Nevertheless, different strategies proved successful [14], also aimed at the formation of catalytic hydrogels as functional materials [15].

**Scheme 1:**
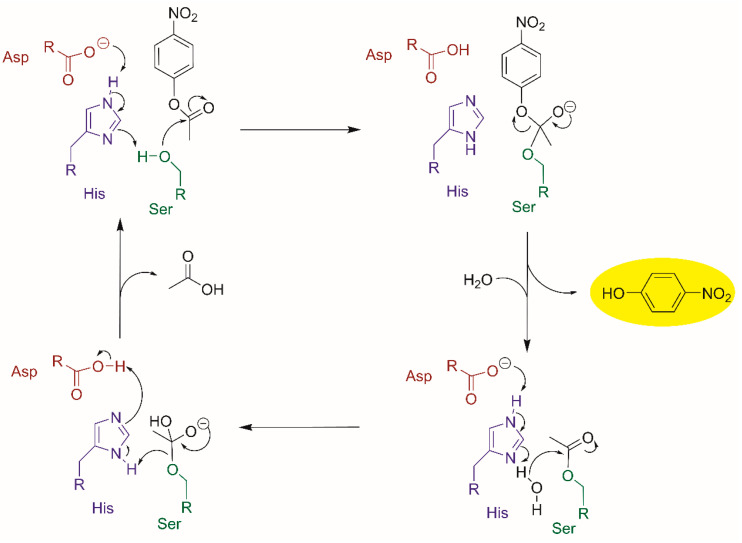
Example of catalytic mechanism of serine proteases for the hydrolysis of 4-nitrophenyl acetate, a common substrate, that leads to the formation of the yellow-colored 4-nitrophenol that can be measured spectrophotometrically.

A very popular approach to achieve a biocatalytic nanostructure for hydrolase mimicry consists of the design of a self-assembling peptide that contains histidine (His) in the sequence. Earlier reports demonstrated that this strategy was successful on several 14-mers [16]. Since then, there has been a continuous effort toward the inclusion of His in small-molecular-weight derivatives as gelators [17] or the gradual shortening of the peptide sequence, for instance to 13-mers [18], 11-mers [19], heptapeptides [20,21], tetrapeptides [22], tripeptides [23,24], or cyclic dipeptides [25]. In other systems, activity could be boosted by the presence of cations, such as zinc [26,27,28,29] or iron [30], or by additional amino acids to mimic the catalytic triad (Scheme 1) [31], such as Ser, [32,33] Arg [16,34], or Asp [35,36]. We recently reported a minimalistic sequence composed of the unprotected, heterochiral tripeptide l-His–d-Phe–d-Phe (or its enantiomer d-His–l-Phe–l-Phe), able to self-organize in phosphate buffer into biocatalytic nanostructures that, at higher concentrations, yielded thermoreversible hydrogels [24]. The design was based on the combination of both d- and l-amino acids at specific positions along a heterochiral sequence [37], so that gelling, amphipathic supramolecular structures could arise due to the diphenylalanine self-assembling motif [38]. When assembled, the tripeptide demonstrated an esterase-like activity on the hydrolysis of 4-nitrophenyl acetate (pNPA), a model compound that got hydrolyzed to the yellow-colored 4-nitrophenol (pNP) [39]. A proposed mechanism involves the presence of both a free base acting as a nucleophile or general base (e.g., the His side chains or amine at the *N*-terminus), as well as a protonated His stabilizing the oxyanion intermediate [40]. Self-assembly leads to a change in the chemical environment around the His residues, and this is a well-known factor [41,42,43] that can induce an apparent pKa shift [44] which could activate the peptide, thus mimicking the role played for instance by Asp in the catalytic triad [41]. An attractive feature of supramolecular peptide-based catalysts is the possibility to recycle the systems, for instance, by means of pH switches to control assembly/disassembly cycles [45]. 

Nevertheless, the de novo design of catalytic supramolecular systems based on minimalistic peptides is still rather challenging, and even minor structural modifications may affect self-assembly and catalysis in ways that are difficult to predict. For instance, C-terminal amidation was previously reported to accelerate the gelation kinetics of Fmoc-Phe derivatives [46], whereas presence of a carboxylic acid moiety at the C-terminus may assist with the catalysis [33]. Therefore, in order to gain new insights into these systems, this work analyzed the supramolecular behavior and catalytic performance of d-His–l-Phe–l-Phe *N*-acetylated, *C*-amidated, or both, with the ultimate aim of gathering useful information for the future design of enhanced systems.

## 2. Results and Discussion

### 2.1. Peptide Self-Assembly into Nanostructured Hydrogels

**Scheme 2:**
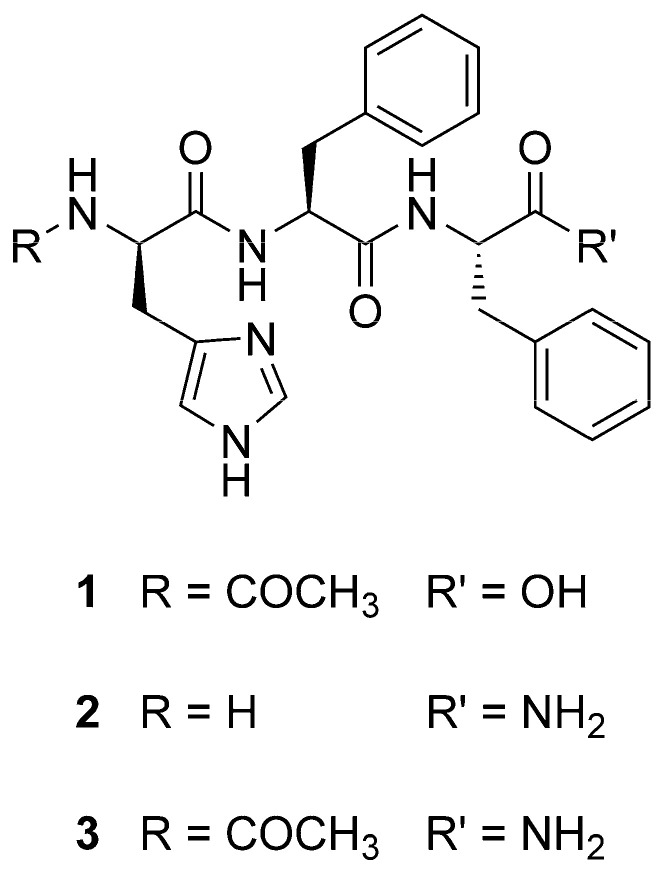
The three tripeptides studied in this work.

**Figure 1:**
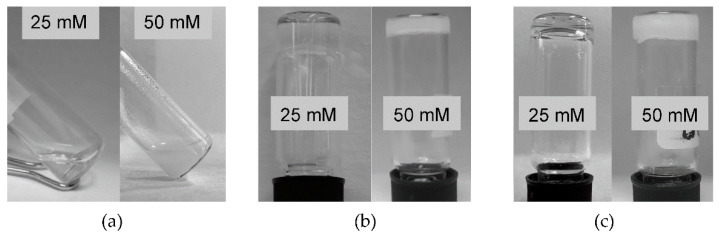
Photographs of (**a**) **1**, (**b**) **2**, and (**c**) **3** in phosphate buffer at 25 and 50 mM.

The peptides shown in Scheme 2 were synthesized in solid phase according to established protocols [47], purified by reverse-phase HPLC, freeze-dried, and characterized by ^1^H- and ^13^C-NMR and ESI-MS to verify their purity and identity (see Supplementary Materials). At neutral pH, the parent compound l-His–d-Phe–d-Phe (or its enantiomer d-His–l-Phe–l-Phe) was shown to stack, through hydrogen bonding between amide groups, into β-sheet-like structures to give rise to amyloid fibrils, which, at higher concentrations, further bundled into fibers and gelled [24]. Analogously, each peptide **1**–**3** was tested for self-assembly and hydrogelation in phosphate buffer at neutral pH. Interestingly, *N*-acetylated **1** did not gel at all, yielding macroscopic aggregates beyond its solubility limit. By contrast, **2** and **3** formed stable hydrogels already at 25 mM (Table 1 and Figure 1). For comparison, the tripeptide analogue with unprotected termini displayed a minimum gelling concentration (mgc) of 50 mM [24]. We inferred that terminus modification increased the compound hydrophobicity and lowered the mgc. HPLC retention times (see Supplementary Materials), which can be considered an experimental measure of hydrophobicity [48], were in the order **2** << **3** ≈ **1**, which can be rationalized considering that **2** is the only one with an ionizable group at the N-terminus. Additionally, hydrogelation is a complex process that requires not only hydrophobicity to drive molecular aggregation, but also the generation of amphipathic architectures, whereby hydrophilic surfaces engage through hydrogen-bonding interactions with water [48]. If compound **1** displayed a deprotonated carboxylate anion at the *C*-terminus at neutral pH, then it would have fewer hydrogen-bonding donors relative to amidated **2** and **3** and, thus, reduced possibilities for interactions with water, which may explain the formation of aggregates as opposed to a hydrogel.

**Figure 2:**
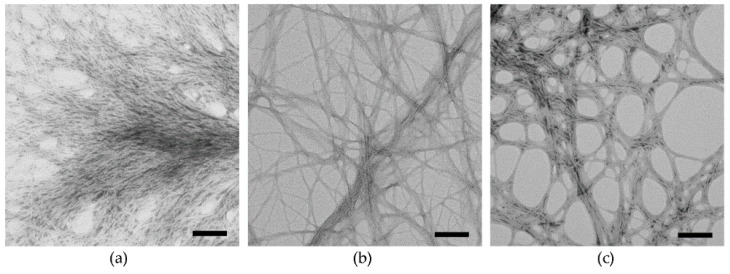
TEM micrographs of (**a**) **1**, (**b**) **2**, and (**c**) **3** nanofibrils (25 mM). Scalebar = 200 nm.

**Figure 3:**
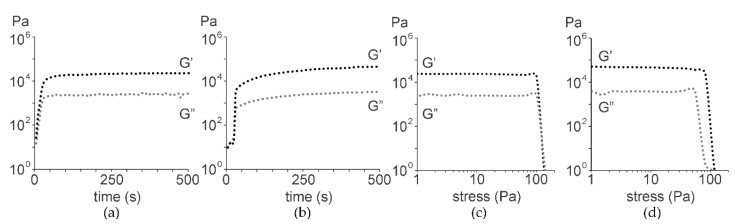
Oscillatory rheometry time sweeps for (**a**) **2** and (**b**) **3**; stress sweeps for (**c**) **2** and (**d**) **3**.

Transmission electron microscopy revealed the presence of nanofibrils in all cases (Figure 2). Interestingly, their diameters were 11 ± 1 nm for **1**, 8 ± 2 nm for **2**, and 11 ± 3 nm for **3** (*n* = 100). Oscillatory rheometry was then used to assess the viscoelastic properties of the resulting materials (Figure 3). Both **2** and **3** gelled within a minute, with gelation kinetics being faster for **2** relative to **3**. A possible explanation lies in the fact that only **2** displays an ionizable N-terminus; thus, following the pH trigger, the ammonium species forms immediately and prompts gelation. By contrast, the ionization state of **3** is not expected to vary upon application of the pH change from alkaline to neutral, and it may, thus, take longer for this species to self-organize into a fibrillar hydrogel. After 1 h, the elastic modulus G’ reached 23 kPa for **2** and 68 kPa for **3**. The higher stiffness for the hydrogel composed of **3**, relative to **2**, was in agreement with the wider fibrils observed by TEM for the former relative to the latter. Stress sweeps revealed a linear viscoelastic region up to ca. 70 Pa for **2** and 30 Pa for **3**. It is possible that the thinner fibrils of **2** displayed higher interconnectivity leading to higher stability against applied stress, as already observed for other gelling heterochiral tripeptides [48].

### 2.2. Peptide Conformational Study

**Figure 4:**
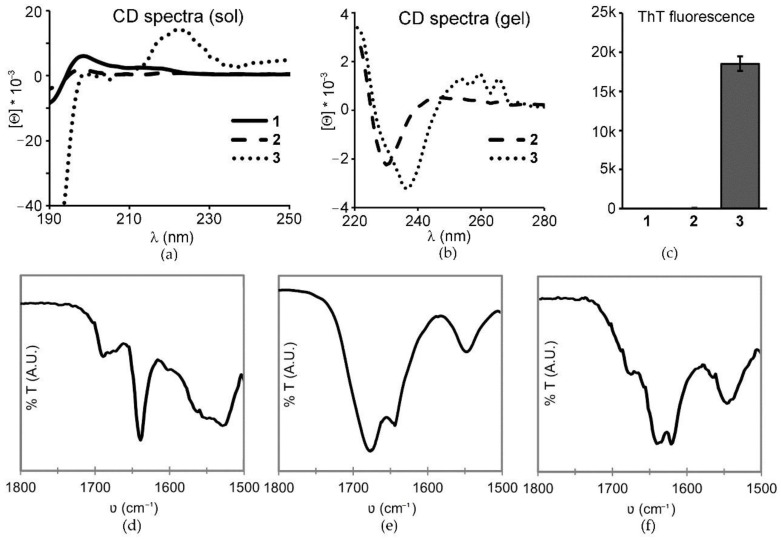
Circular dichroism (CD) spectra in the sol (**a**) and gel (**b**) states; (**c**) thioflavin T fluorescence; (**d**–**f**) Fourier-transform infrared (FT-IR) spectra of compounds **1** (**d**), **2** (**e**), and **3** (**f**) in the gel state.

Circular dichroism (CD) spectra were acquired in the sol state (1 mM) for each compound (Figure 4a). **1** and **2** displayed very weak signals; however, the overall signature with two maxima at 199 and 219 nm was analogous to the one previously observed for similar assembling heterochiral tripeptides [48]. In that case, a combination of experimental and in silico data allowed deciphering the CD spectra as a population of conformations in solution, of which the most stable populated the top-left quadrant of the Ramachandran plot, where β-structures (sheets and turns) are located. By contrast, the spectrum of **3** was dissimilar and displayed a negative signal under 200 nm, and a positive maximum centered at 223 nm, with visible scattering occurring at higher wavelengths. This signal was already reported for similar short peptides that displayed Phe–Phe interactions and may be indicative of compound **3**’s marked tendency to stack into fibrils that yield scattering [49]. CD data were also acquired at gelling concentrations (Figure 4b), although, in this case, acquisition was possible only above 220 nm due to high voltage. Interestingly, assembly significantly altered the spectra for both **2** and **3** since the gels displayed a negative minimum at 230 and 238 nm, respectively. Furthermore, the gel of **3** displayed the typical vibronic signature for the forbidden π–π* transition of the aromatic side chains of Phe, as a result of its inclusion in a supramolecular chiral environment, noted also for the parent compound [24]. 

Thioflavin T fluorescence (ThT) was then used as an assay to determine the presence of amyloid structures [50], since the dye is known to bind onto hydrophobic grooves of the surface of fibrils composed of at least four consecutive β-sheets [51]. As a result of the binding, the rotation is impeded between the two aromatic rings that compose the dye, yielding fluorescence [52]. Surprisingly, only compound **3** displayed marked fluorescence (Figure 4c), despite the fact that all three formed nanofibrils as evidenced by TEM. Clearly, the surface topography of the fibrils composed of **3** was different relative to the other analogues, and it bound thioflavin more efficiently. It has been shown that ThT binding is not mediated by electrostatic interactions, and it is, thus, favored on fibrils formed by neutral species as is compound **3**, and disfavored onto highly charged surfaces, as may be those of compounds **1** and **2 [53]**. The amide I region of attenuated total reflectance (ATR) Fourier-transformed infrared (FT-IR) spectra of the three gels (Figure 4d–f) revealed a major peak at 1638 cm^−1^ for **1** and **3**, which is typical for β-sheet-like structures; this signal was shifted to 1645 cm^−1^ in the case of compound **2**. Compound **3** was the only one to also display an intense signal at 1620 cm^−1^, which is in the region of amyloid structures, in agreement with the ThT fluorescence data. Additionally, all spectra presented a broad signal in the region of 1670–1680 cm^−1^ that could be ascribed to the presence of residual trifluoroacetate.

Interestingly, when the hydrogels formed by **2** or **3** were tested against heating in a water bath, both started to disassemble at rather high temperature values (i.e., 65 °C for **2** and 70 °C for **3**), but only **2** was thermoreversible and reformed upon cooling to room temperature. By contrast, **3** expelled water and irreversibly formed aggregates.

### 2.3. Esterase-Like Biocatalysis

Having confirmed that all three compounds self-assembled at neutral pH into fibrils, they were tested as biocatalysts for esterase mimicry. 4-Nitrophenyl acetate (pNPA) was chosen as a model compound, since it gets hydrolyzed to the yellow-colored 4-nitrophenol that can easily be monitored spectrophotometrically [39]. The three compounds in solution at 1 mM did not show significant catalysis, analogously to the parent heterochiral compound His–Phe–Phe [24]. The observed reaction rates with 1 mM pNPA corresponded to 4.43 × 10^−5^ s^−1^ (**1**), 2.00 × 10^−5^ s^−1^ (**2**), and 3.85 × 10^−5^ s^−1^ (**3**). Under self-assembly conditions, the thermoreversible hydrogel formed by C-amidated **2** displayed the best catalytic performance, corresponding to a k_obs_ of 8.68 × 10^−3^ s^−1^ at 50 mM, which surpassed that of the parent heterochiral His–Phe–Phe with free termini under analogous conditions (1.7 × 10^−3^ s^−1^) [24], thus suggesting that a free carboxylic acid at the C-terminus did not significantly assist in catalysis. In the same conditions, compound **1** formed aggregates, whose performance was worse than the fibrils obtained at the lower concentration of 25 mM (Table 2). Compared to the parent compound, the catalytic performance under analogous conditions in the fibril state was slightly worse, suggesting a positive role of the free N-terminus in catalysis. Lastly, compound **3** displayed the worst biocatalytic activity of the set of three. For comparison, under analogous conditions relative to those for the best catalytic performance of **1** (bold in Table 1), **3** displayed a k_obs_ of 4.55 × 10^−4^ s^−1^. It should be noted that it was not possible to analyze the catalytic performance of this compound at 50 mM due to a high level of scattering from the white gel. Overall, the data indicated that *N*-acetylation negatively affected biocatalysis, while *C*-amidation alone improved it.

In conclusion, *N*-acetylation and/or *C*-amidation were shown to affect the self-assembly ability and, to a minor extent, the biocatalytic performance of the heterochiral His–Phe–Phe tripeptide [24]. All three compounds self-organized into nanofibrils, although the *N*-acetylated analogue **1** did not gel at all, and the *N*-acetylated and *C*-amidated **3** formed irreversible hydrogels. We infer that the presence of fewer hydrogen-bonding donor groups in **1** relative to the other compounds negatively affected its ability to interact with water to gel. Furthermore, compounds **2** and **3** displayed an mgc corresponding to 25 mM, in contrast with 50 mM for the parent compound, suggesting that terminal modification increased hydrophobicity and favored hydrogelation. In particular, *C*-amidated **2** was the only compound of the set to maintain the ability to form thermoreversible hydrogels, with an improvement in catalytic performance. These data confirmed that the presence of a free carboxylic acid at the *C*-terminus did not significantly assist in the catalysis performed by this tripeptide sequence. As an additional value, recent reports described a positive effect of *C*-amidation on the stability and biological performance of short heterochiral peptides [36]. Future studies will focus on other strategies to enhance the catalytic activity of the His–Phe–Phe sequence, for instance, through co-assembly with other peptides bearing different amino acids, such as Ser or Arg, which could mimic the catalytic triad found in hydrolases, or through peptide sequence extension to include the same residues. For instance, addition of Arg units at the termini of short self-assembling peptides was shown to reduce hierarchical association of fibrils and improve gelation kinetics [37].

## 3. Materials and Methods

### 3.1. Materials and General Methods

All chemicals and solvents were purchased of analytical grade from Merck (Milan, Italy), except for the reagents used for peptide synthesis, which were purchased from GL Biochem (Shanghai, China). Peptides were synthesized in solid phase using standard protocols based on Fmoc-protection and HBTU/HOAt activation [47]. Purification was performed on reverse-phase HPLC using an Agilent 1260 Infinity system, equipped with a C-18 column (Kinetex, 5 microns, 100 Å, 250 × 10 mm, Phenomenex). The gradient used consisted of acetonitrile/water with 0.05% TFA with the following program: t = 0–2 min, 25% MeCN; t = 14–16 min, 95% MeCN. The purified fractions were freeze-dried to yield a white, fluffy powder. ESI-MS characterization was performed on an Agilent 6120 Infinity (Agilent Technologies, Milan, Italy), and NMR spectra were acquired on a Varian Inova (Varina Inc., Milan, Italy (further details are in the Supplementary Materials). MilliQ water was obtained with an inline Millipore RiOs/Origin system (Merck, Milan, Italy) with resistivity higher than 18 MΩ∙cm. All experiments were performed with each peptide at various concentrations in sodium phosphate buffer 0.1 M with a final pH of 7.1 ± 0.1 following the protocol. Self-assembly was tested by dissolving each compound in 0.1 M sodium phosphate (pH 11.8), followed by mild heating if needed to aid with dissolution to achieve a final pH of 7.1 ± 0.1. Compound **3** could not be dissolved following this protocol at 50 mM; therefore, only in this case, the peptide was dissolved in half of the final volume using 0.1 M sodium phosphate buffer at pH 5.8, followed by dilution with an equal volume of 0.1 M sodium phosphate buffer at pH 11.8 to reach the final pH of 7.1 ± 0.1. In the case of peptide solutions at 1 mM, each peptide was dissolved in half of the final volume consisting of 0.1 M sodium phosphate buffer at pH 11.8, followed by dilution with an equal volume of sodium phosphate 0.1 M at pH 5.8 to reach neutrality as described above. Thermoreversibility was assessed by immersing the vials shown in Figure 1 in an oil bath that was heated at 5 °C/min until ca. 80 °C, in which condition no gel residue was left, and samples were left to cool down to room temperature.

### 3.2. Oscillatory Rheology

Hydrogels composed of **2** or **3** were formed at 25 mM in situ in an oscillatory rheometer Kinexus Ultra Plus (Malvern, Alfatest, Milan, Italy) using a stainless-steel 20 mm parallel plate geometry at 25 °C. Time sweeps were recorded at a frequency of 1 Hz and a stress of 5 Pa. Stress sweeps were recorded at 1 Hz. Measurements were repeated twice on independent experiments and a representative dataset is shown.

### 3.3. Transmission Electron Microscopy

TEM micrographs were acquired using a Philips electron microscope 208 (FEI, Hillsboro, Oregon, OR, USA) that was equipped with a Quemesa (Olympus Soft Imaging Solutions (Berlin, Germany) camera; images were recorded with RADIUS software; samples were prepared as described previously using phosphotungstate as a negative stain [24].

### 3.4. Circular Dichroism (CD)

CD spectra were recorded in a J-815 system (Jasco, Easton, MD, USA) using a 0.1 mm quartz cuvette at 25 °C, with a bandwidth of 1 nm and a step size of 1 nm with 1 s integration. Spectra shown are the average of at least five measurements (one accumulation each).

### 3.5. Thioflavin T Fluorescence

Samples (0.12 mL) were prepared as described above at 25 mM peptide concentration and immediately put on wells of Greiner 96 Flat Clear-Bottom Black Polystyrene. After 4 h, 30 μL of a Thioflavin T solution (22.2 μM in 20 mM glycine-NaOH pH 8.5, 0.2 μm filtered) was added to the wells. After 15 min, fluorescence was analyzed using a Tecan Infinite M1000 Pro (Tecan, Milan, Italy), with an excitation wavelength of 446 nm, an emission wavelength of 490 nm, and a bandwidth of 20 nm. Each condition was repeated twice in triplicate. Average and standard deviations were calculated and plotted with Excel.

### 3.6. ATR FT-IR Spectroscopy

Hydrogel samples (50 mM) were prepared as described above for compounds **1**–**3**, and, after 1 h of self-assembly, they were placed atop a small piece of Silicon wafer and dried in vacuo overnight. IR spectra were then acquired on an Affinity-1S (Shimadzu, Milan, Italy), diamond, 4 cm^−1^ resolution, 240 scans).

### 3.7. Biocatalysis

Samples were prepared as described above, and 98 μL were immediately put in wells of Greiner 96 U Bottom Transparent Polystyrene. Compounds **1**–**3** were tested at 1 mM, as well as at higher concentrations. In particular, compound **1** was tested at 25 and 50 mM. Hydrogels of **2** were tested at 50 mM, while hydrogels of **3** could not be tested at 50 mM due to scattering; hence, they were probed at 25 mM. After 4 h, 2 μL of a solution of pNPA [24] (10 mM in MeOH) was added to the wells, unless otherwise stated (i.e., for more diluted conditions, pNPA concentration was lowered accordingly). Absorbance at 405 nm was monitored over 1 h on a Tecan Infinite M1000 Pro (Tecan, Milan, Italy). Each condition was repeated twice in triplicate. K_obs_ was calculated as previously described [24].

## 4. Conclusions

*N*-acetylation and/or *C*-amidation were shown to affect the self-assembly ability and, to a minor extent, the biocatalytic performance of the heterochiral His–Phe–Phe tripeptide [24]. All three compounds self-organized into nanofibrils, although the *N*-acetylated analogue **1** did not gel at all, and the *N*-acetylated and *C*-amidated **3** formed irreversible hydrogels. We infer that the presence of fewer hydrogen-bonding donor groups in **1** relative to the other compounds negatively affected its ability to interact with water to gel. Furthermore, compounds **2** and **3** displayed an mgc corresponding to 25 mM, in contrast with 50 mM for the parent compound, suggesting that terminal modification increased hydrophobicity and favored hydrogelation. 

In particular, *C*-amidated **2** was the only compound of the set to maintain the ability to form thermoreversible hydrogels, with an improvement in catalytic performance. These data confirmed that the presence of a free carboxylic acid at the *C*-terminus did not significantly assist in the catalysis performed by this tripeptide sequence. As an additional value, recent reports described a positive effect of *C*-amidation on the stability and biological performance of short heterochiral peptides [36]. Future studies will focus on other strategies to enhance the catalytic activity of the His–Phe–Phe sequence, for instance, through co-assembly with other peptides bearing different amino acids, such as Ser or Arg, which could mimic the catalytic triad found in hydrolases, or through peptide sequence extension to include the same residues. For instance, addition of Arg units at the termini of short self-assembling peptides was shown to reduce hierarchical association of fibrils and improve gelation kinetics [37].

## Figures and Tables

**Table 1:** Hydrogels were formed by compounds **2** and **3**, but not **1**, in phosphate buffer at neutral pH.

Tripeptide	N-Terminus	C-Terminus	1 mM	25 mM	50 mM
l-His–d-Phe–d-Phe	NH_2_	COOH	Sol	Sol	Hydrogel
**1**	Acetylated	COOH	Sol	Sol	Aggregates
**2**	NH_2_	Amidated	Sol	Hydrogel	Hydrogel
**3**	Acetylated	Amidated	Sol	Hydrogel	Hydrogel

**Table 2:** Catalytic rates (k_obs_, s^−1^) of peptides **1**–**3** on the hydrolysis of 4-nitrophenyl acetate (pNPA). The best performance for each peptide is indicated in bold.

[pNPA] mM	1 (Sol)	1 (Fibrils)	1 (Aggregates)	2 (Hydrogel)	3 (Hydrogel)
0.2	1.02 × 10^−^^5^	2.17 × 10^−4^	1.05 × 10^−4^	3.55 × 10^−4^	3.83 × 10^−5^
0.4	1.65 × 10^−5^	4.39 × 10^−4^	2.00 × 10^−4^	2.60 × 10^−3^	1.50 × 10^−4^
0.6	2.43 × 10^−5^	7.61 × 10^−4^	3.57 × 10^−4^	5.00 × 10^−3^	3.11 × 10^−4^
0.8	3.02 × 10^−5^	9.70 × 10^−4^	4.32 × 10^−4^	7.26 × 10^−3^	4.41 × 10^−4^
1.0	4.43 × 10^−5^	**1.16 × 10^−3^**	6.60 × 10^−4^	**8.68 × 10^−3^**	**4.55 × 10^−4^**

## Data Availability

Data is contained within the article or Supplementary Material.

## Supplementary Materials

The following are available online. ^1^H- and ^13^C-NMR spectra, ESI-MS spectra, HPLC traces, and biocatalytic data of compounds **1**–**3**.

## Sample Availability

Samples of the compounds **1**–**3** are available from the authors.
