# Supplementary material for: Tripeptide Self-Assembly into Bioactive Hydrogels: Effects of Terminus Modification on Biocatalysis
Source: Molecules. 2020 Dec 31;26(1):173. doi: 10.3390/molecules26010173 (PMC7794889; doi:10.3390/molecules26010173)
Supplement: Supplementary file 1 [file molecules-26-00173-s001.pdf]

Supporting Information

Tripeptide self-assembly into bioactive hydrogels: effects of terminus modification on biocatalysis

Marina Kurbasic[1], Ana M. Garcia[1], Simone Viada[1], and Silvia Marchesan[1]*

[1]Chemical & Pharmaceutical Sciences Department, University of Trieste, Trieste 34127, Italy; marina.kurbasic@studenti.units.it (M.K.); anamariagarcia.1988@gmail.com (A.M.G); simone.viada@studenti.units.it (S.V.), smarchesan@units.it (S.M.).

Table of Contents

1. Spectroscopic data for compound **1**... 2
2. Spectroscopic data for compound **2**... 4
3. Spectroscopic data for compound **3**... 6
4. HPLC traces for compounds **1-3**... 8
5. Catalysis data for compounds **1-3**... 9

Spectroscopic data for compound **1**

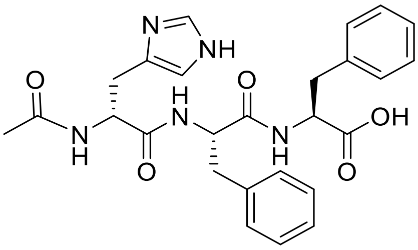

^{1}**H NMR** (400 MHz, DMSO-d_6) δ 8.84 (s, 1H, CH His), 8.40 (d, *J* = 7.9 Hz, 1H, NH), 8.18 (d, *J* = 8.7 Hz, 1H, NH), 8.03 (d, *J* = 8.4 Hz, 1H, NH), 7.24 – 7.19 (m, 10H, Ar), 6.85 (s, 1H, CH His), 4.57 – 4.51 (m, 2H, αCH), 4.46-4.40 (m, 1H, αCH), 3.10 – 2.88 (m, 3H, βCH_2), 2.80-2.75 (dd, *J* = 15.2, 5.4 Hz, 1H, βCH_2), 2.69-2.57 (m, 2H, βCH_2), 1.77 (s, 3H, CH_3). ^{13}**C NMR** (100 MHz, DMSO-d_6) δ (ppm) 173.1 (COOH); 171.4, 170.0, 169.9 (3 x CO); 158.7, 158.37, 138.0, 137.9, 134.1, 129.6, 129.6, 128.7, 128.4, 126.9, 126.7, 119.0, 116.75, 116, 110.0 (Ar); 54.0, 53.9, 51.8 (3 x αC); 38.0, 37.0, 27.7 (3 x βC); 22.9 (CH_3). **ESI-MS** m/z 492.2 $(M+H)^+$ $C_{26}H_{30}N_5O_5$ requires 492.2.

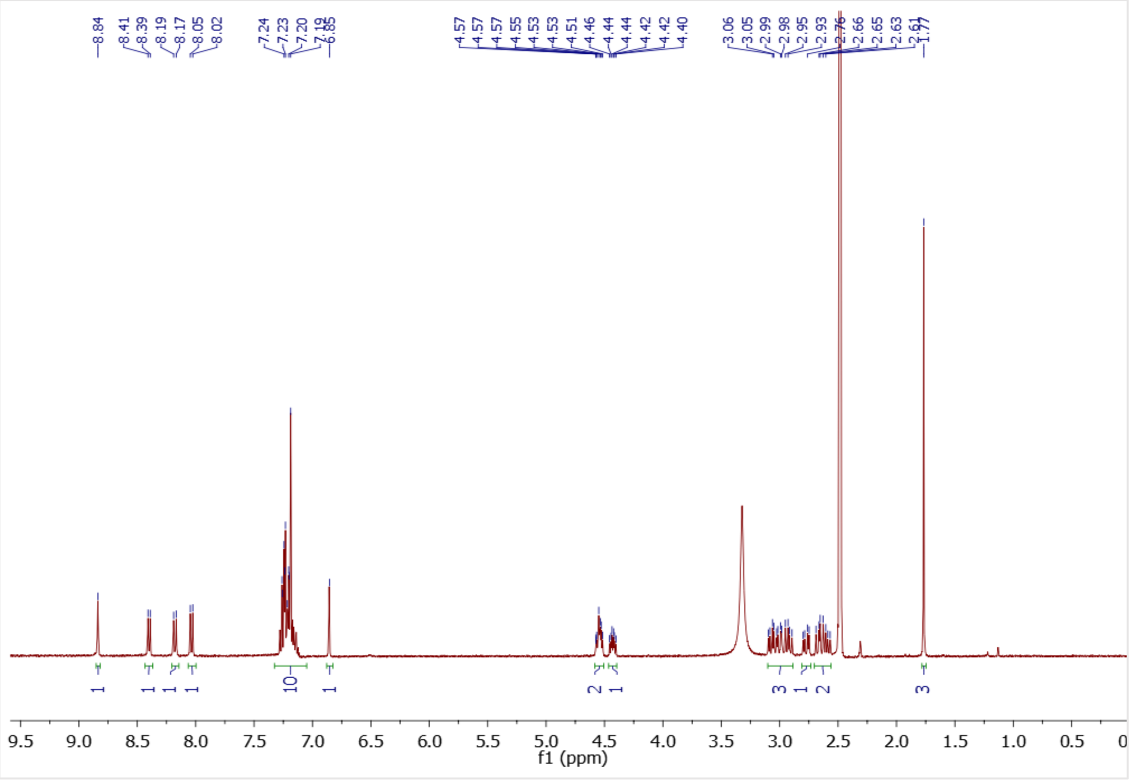

Figure S1. ^{1}H-NMR spectrum of **1**.

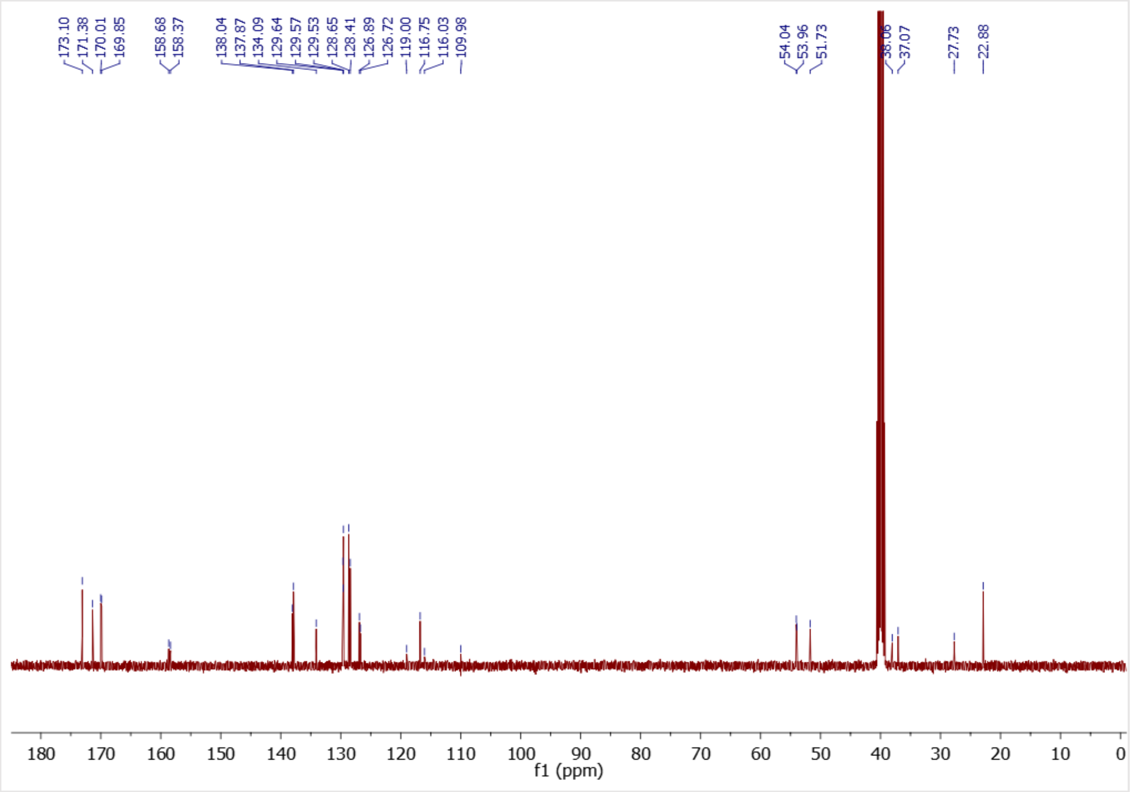

Figure S2. ^{13}C-NMR spectrum of **1**.

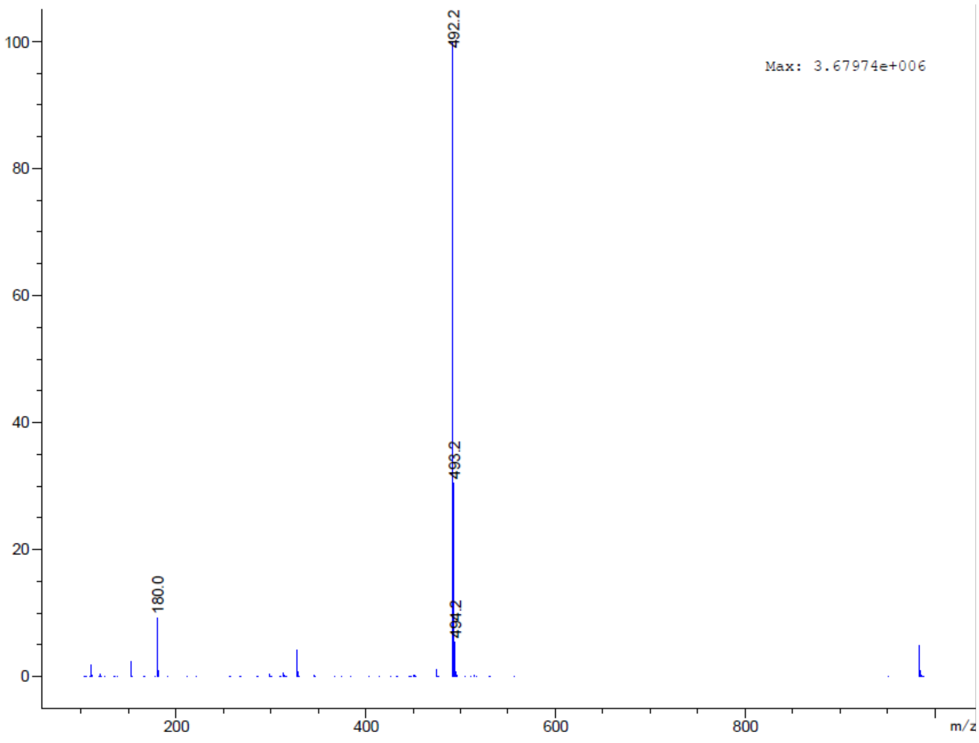

Figure S3. ESI-MS spectrum of **1**.

Spectroscopic data for compound **2**

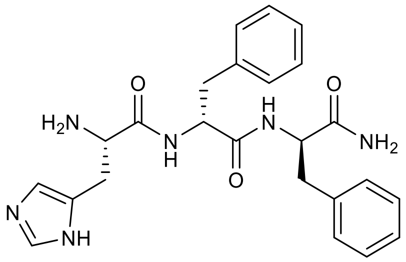

^{1}H NMR (400 MHz, DMSO-d_6) δ (ppm) 8.89 (s, 1H, NH), 8.68 (d, J = 8.7 Hz, 1H, NH), 8.47 (d, J = 8.2 Hz, 1H, NH), 8.15 (s, 3H, NH_3^+), 7.44 (s, 1H, H-ar), 7.32 – 7.03 (m, 10H, Ar), 6.89 (s, 1H, H-ar), 4.76 – 4.64 (m, 1H, αCH), 4.46 (td, J = 8.7, 5.1 Hz, 1H, αCH), 4.15 – 4.01 (m, 1H, αCH), 3.06 (dd, J = 3.9 Hz, *Jgem* = 13.8 Hz, 1H, $βCH_2$), 3.01 ((dd, J = 5.1 Hz, *Jgem* = 13.8 Hz, 1H, $βCH_2$), 2.90 ((dd, J = 4.7 Hz, *Jgem* = 15.8 Hz, 1H, $βCH_2$), 2.82 (dd, J = 9.2 Hz, *Jgem* = 13.9 Hz, 1H, $βCH_2$), 2.70 (dd, J = 7.4 Hz, *Jgem* = 15.2 Hz, 1H, $βCH_2$). **^{13}C NMR** (100 MHz, DMSO-d_6) δ (ppm) 173.1, 170.9, 167.3 (3 x CO); 138.3, 137.7, 134.8, 129.7, 129.6, 128.6, 128.4, 126.8, 119.0, 117.3, 115.9 (Ar); 54.6, 54.1, 51.6 (3 x αC); 38.7, 38.2, 26.8 (3 x βC). **ESI-MS** m/z 449.2 $(M+H)^+$ $C_{24}H_{28}N_6O_3$ requires 449.2.

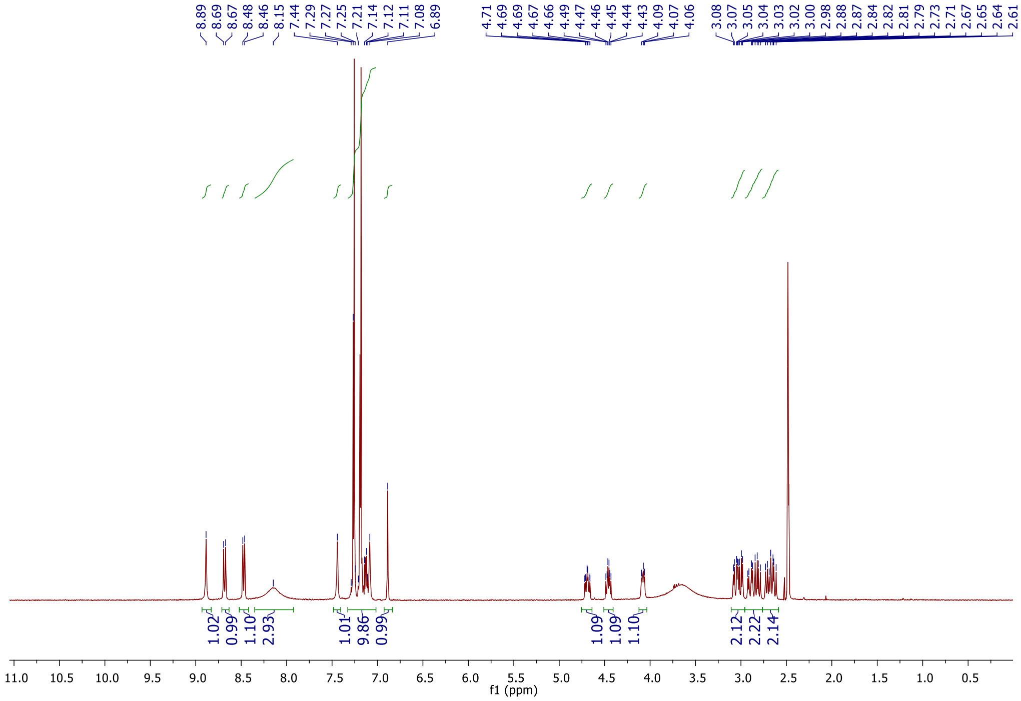

Figure S4. ^{1}H-NMR spectrum of **2**.

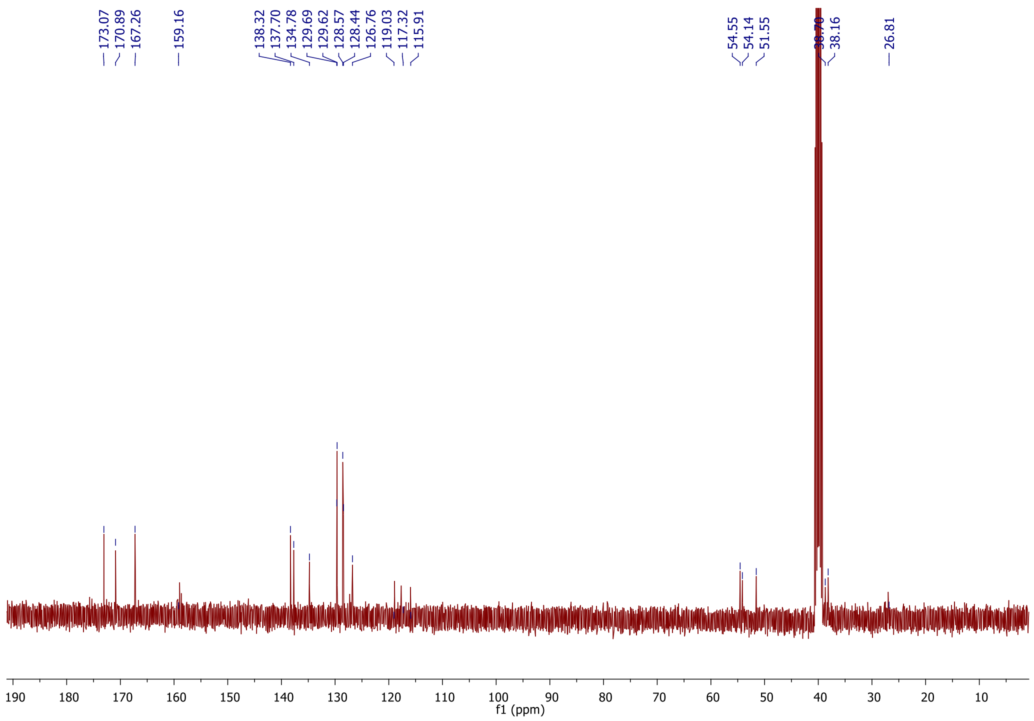

Figure S5. ^{13}C-NMR spectrum of **2**.

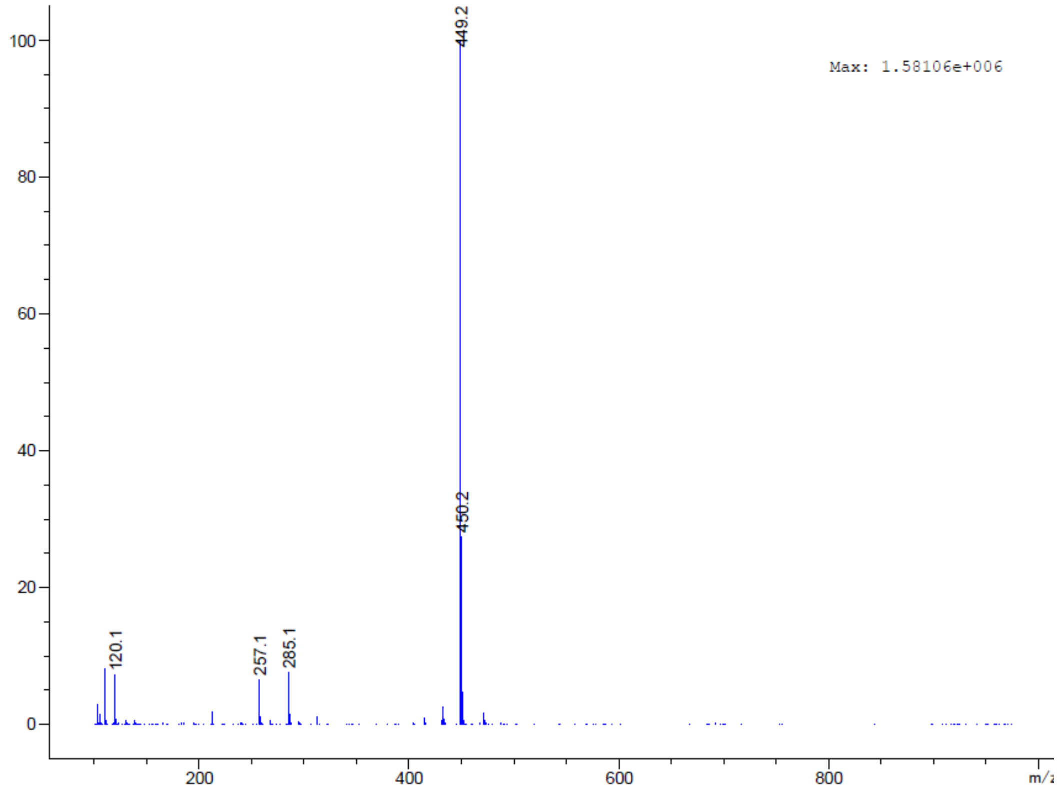

Figure S6. ESI-MS spectrum of **2**.

Spectroscopic data for compound **3**

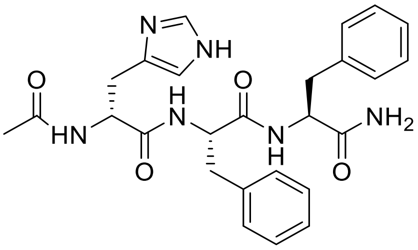

^{1}H NMR (400 MHz, DMSO-d_6) δ (ppm) 8.90 (d, J = 1.3 Hz, 1H, CH sp^2), 8.19 (d, J = 8.2 Hz, 1H, NH), 8.07 (d, J = 8.3 Hz, 1H, NH), 7.96 (d, J = 7.9 Hz, 1H, NH), 7.30 (s, 1H, NH_2), 7.26 – 7.14 (m, 11H, Ar + NH_2), 7.08 (s, 1H, CH sp^2), 4.55 – 4.40 (m, 3H, αCH), 3.02 – 2.91 (m, 3H, βCH_2), 2.85 – 2.72 (m, 3H, βCH_2), 1.76 (s, 3H, CH_3). **^{13}C NMR** (100 MHz, DMSO-d_6) δ (ppm) 173.1, 171.0, 170.5, 170.1 (4 x CO); 158.5, 158.4, 138.3, 138.1, 134.1, 129.6, 129.5, 128.5, 128.4, 126.7, 126.7, 119.2, 116.8, 116.2, 108.0 (Ar); 54.5, 54.4, 52.0 (3 x αC); 37.9, 37.8, 27.6 (3 x βC), 22.9 (CH_3). **ESI-MS** m/z 491.2 $(M+H)^+$ $C_{27}H_{30}N_6O_4$ requires 491.2.

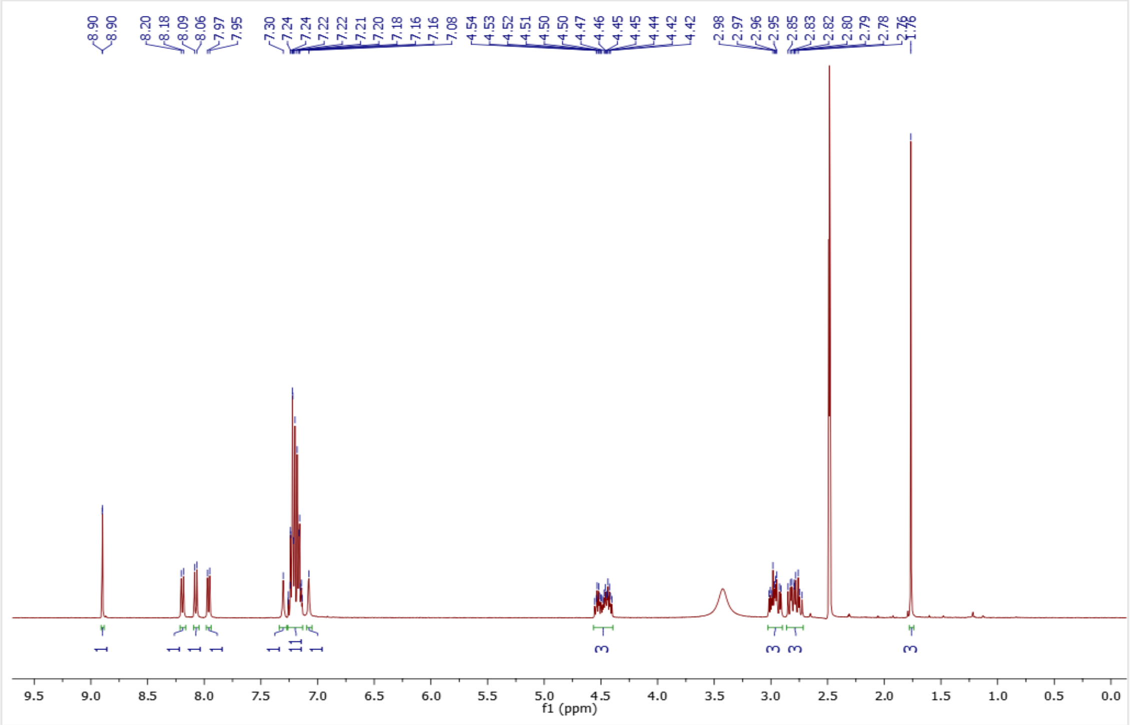

Figure S7. ^{1}H-NMR spectrum of **3**.

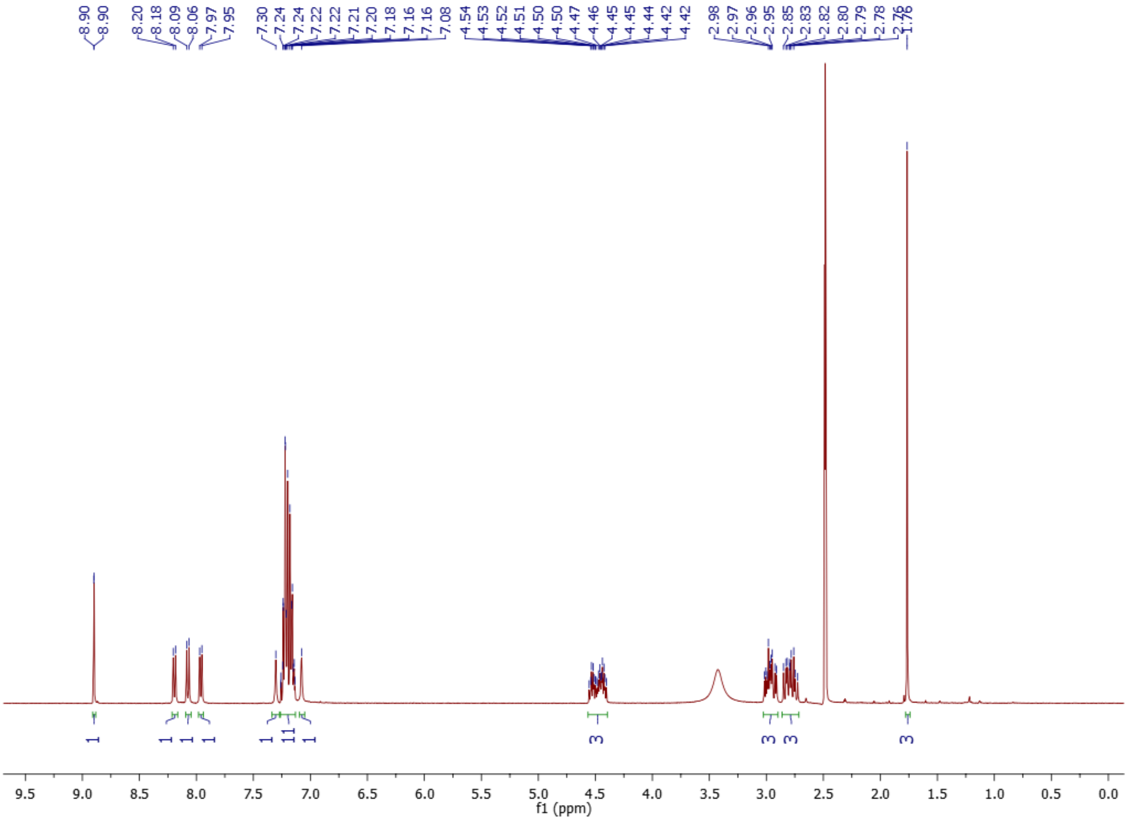

Figure S8. ^{13}C-NMR spectrum of **3**.

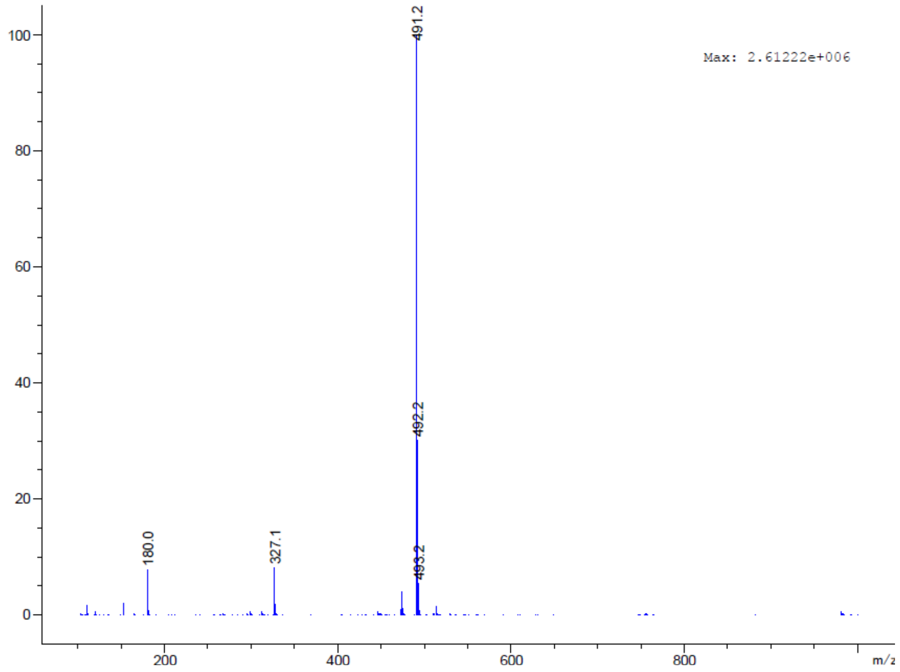

Figure S9. ESI-MS spectrum of **3**.

HPLC traces for compounds **1-3**

Compound **1** R_t = 8.7 min.

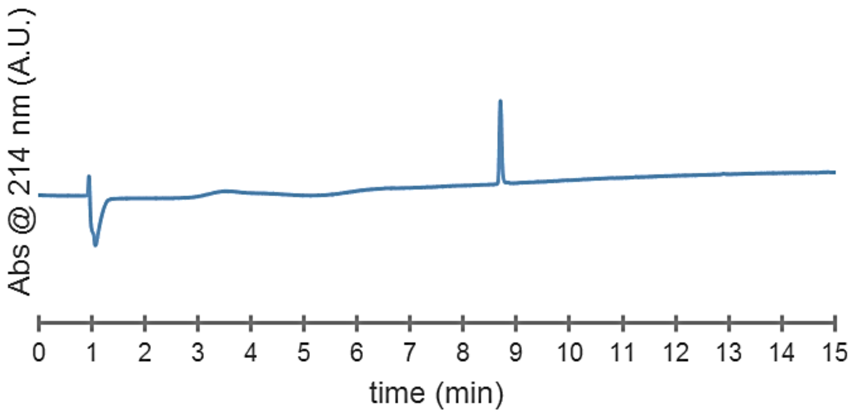

Compound **2** R_t = 5.0 min.

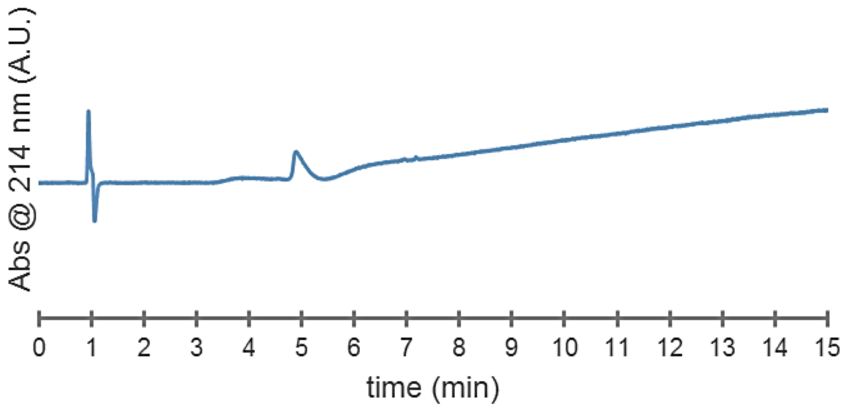

Compound **3** R_t = 8.3 min.

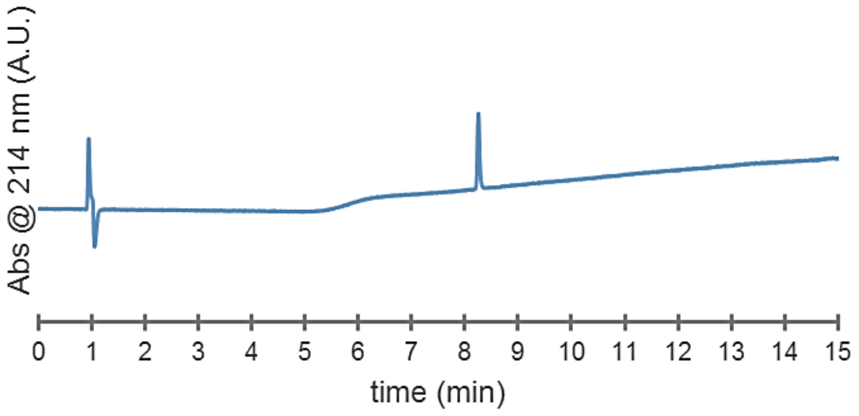

Figure S10. HPLC traces for compounds **1-3**.

Catalysis data for compounds **1-3**

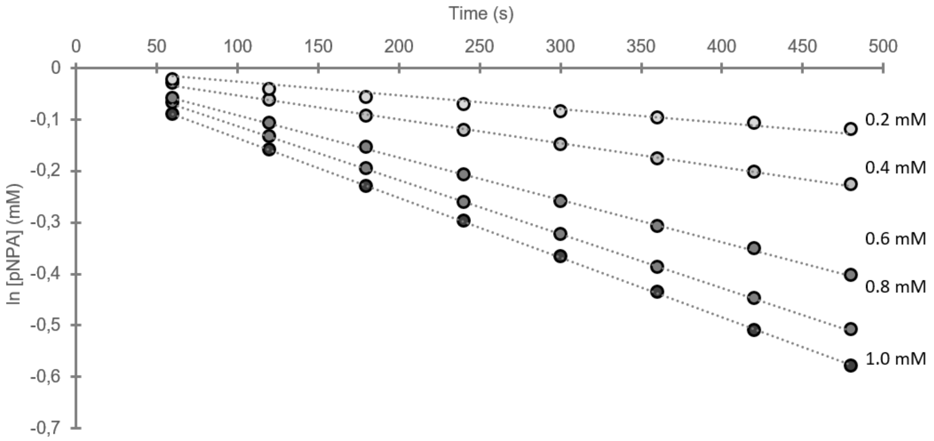

Figure S11. Compound **1** (25 mM) at increasing concentrations of pNPA (0.2-1.0 mM).

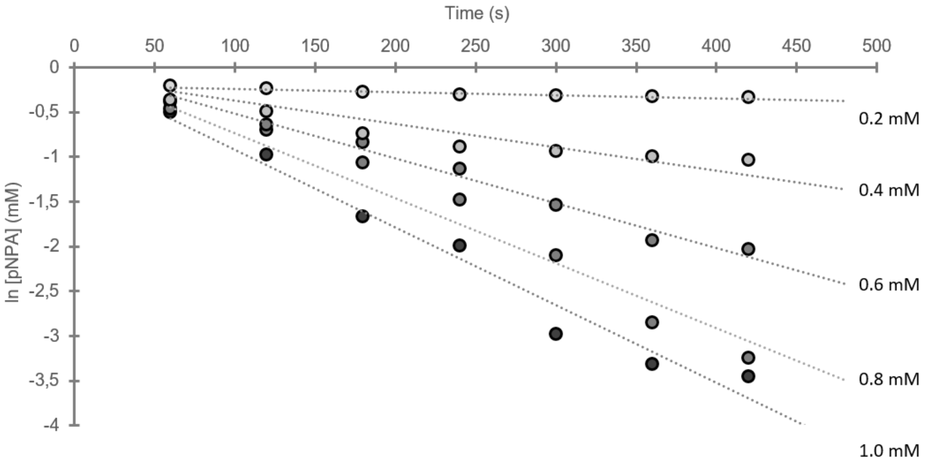

Figure S12. Compound **2** (50 mM) at increasing concentrations of pNPA (0.2-1.0 mM).

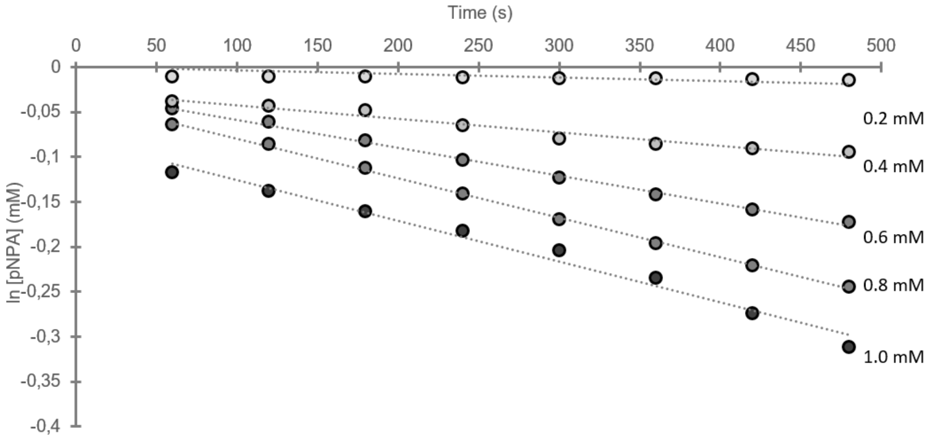

Figure S13. Compound **3** (25 mM) at increasing concentrations of pNPA (0.2-1.0 mM).
